# Integrating technology into cognitive behavior therapy for adolescent depression: a pilot study

**DOI:** 10.1186/s12991-015-0077-8

**Published:** 2015-11-03

**Authors:** Kenneth A. Kobak, James C. Mundt, Betsy Kennard

**Affiliations:** Center for Telepsychology, 22 North Harwood, Madison, WI 53717 USA; UT Southwestern Medical Center, 5323 Harry Hines Boulevard, Dallas, TX 75390 USA

**Keywords:** Cognitive therapy, Internet, Training, Depressive disorder, Adolescent, Dissemination, Evidence based

## Abstract

**Background:**

Rapid advances in information technology and telecommunications have resulted in a dramatic increase in the use of mobile devices and the internet to enhance and facilitate access to treatment. Cognitive behavior therapy (CBT) is an empirically based treatment that is well suited for enhancement by new technologies, particularly with youth. To facilitate the dissemination of this evidence-based treatment, we developed a technology-enhanced CBT intervention for the treatment of adolescent depression consisting of (1) online therapist training (2) in-session use of tablets for teaching clients CBT concepts and skills, and (3) text messaging for between session homework reminders and self-monitoring.

**Methods:**

Eighteen licensed clinicians (social workers *n* = 7, psychologists *n* = 9) were randomized to have their patients receive either the intervention (CBT) or treatment as usual (TAU). Each clinician treated four adolescents for 12 weeks. Clinicians in the CBT arm completed an online tutorial on CBT treatment of adolescent depression, then received an iPad with access to patient education materials for teaching CBT concepts to patients during sessions. Individualized text messages were integrated into treatment for homework reminders, support, and outcomes measurement. Outcome measures included a 49-item multiple choice test for tutorial effectiveness; the system usability scale (SUS) for user satisfaction; quick inventory of depressive symptomatology–adolescent version (QIDS-A-Pat); and clinician and patient ratings on the therapeutic alliance scale for adolescents (TASA).

**Results:**

A significant increase in knowledge of CBT concepts was found after completing the tutorial, *t*(8) = 7.02, *p* < 0.001. Clinician and patient ratings of user satisfaction were high for both the iPad teaching tools, and the text messaging. Ninety-five percent of teens said reviewing their text messages with their therapist was helpful, and all said they would use text messaging in treatment again. Ratings of the therapeutic alliance were higher in the CBT arm *t*(131) = 4.03, *p* = 0.001. A significant reduction in depression was found in both groups [*t*(34) = 8.453, *p* < 0.001 and *t*(29) = 6.67, *p* < 0.001 for CBT and TAU, respectively). Clinical ratings of improvement were greater on all outcome measures for the CBT arm; however, none reached statistical significance. Effect sizes (Cohen’s d) ranged from small (QIDS-A) to large (TASA).

**Conclusions:**

Results support the feasibility of this technology-enhanced CBT intervention as a means of improving CBT treatment of adolescent depression and may help address the critical shortage of therapists trained on empirically based treatments.

## Background

The use of technology for the psychological treatment of mental disorders is on a rapid ascent. While the potential ways of using technology to enhance treatment have been discussed for decades [1, 2], the recent explosion in information technology and telecommunications, and the widespread use of mobile devices have resulted in a dramatic increase in the use of both mobile devices and the internet to enhance and facilitate access to treatment. Several review articles have been published summarizing the bourgeoning body of data being generated [3–7]. Results have generally been supportive of both efficacy and feasibility, though several issues have been identified, such as confidentiality, privacy, crisis management, technological competence, and ethical issues [3, 8]. As with all innovations, new practice guidelines have been developed to address the unique challenges presented [9–11].

Cognitive behavior therapy (CBT) is an empirically based treatment that is uniquely suited to enhancement by new technologies [5, 12]. It is highly structured, typically manualized, follows a sequential progression, emphasizes self-responsibility, self-monitoring and homework, and includes ongoing outcome measurements. A variety of technology-enhanced CBT applications across a range of mental disorders have been reported. These include computer-administered CBT self-treatment (stand alone, no therapist contact), computer-assisted CBT treatment (computer-administered with some clinician guidance or contact), mobile monitoring and communication, psychoeducation, remote live treatment via videoconference, and online therapist training [12–18].

The use of technology is particularly well suited for psychological interventions with youth and teens. Nine in ten teens in the USA (93 %) have access to a computer, 78 % have cell phones, and 74 % have mobile access to the internet via a cell phone, tablet or other device [19]. Text messaging has become the preferred mode of communication among teens, with two-thirds reporting they are more likely to use their cell phones to text their friends than to talk with them. Half of teens in the USA send 50 or more texts per day [20]. Mobile phone use by teens cuts across socio-demographic backgrounds, as more US families replace traditional land lines with mobile phones (e.g., 41 % of households have only wireless according to a 2013 survey by the National Center for Health Statistics; among poor households, the figure is 56 %) [21]. Teens in both the USA and abroad have both the technical expertise with these technologies, and a favorable attitude toward their use in mental health care [4, 22]. Three quarters of lifetime mental disorders begin in adolescence and young adulthood, making it a critical target age for prevention and intervention efforts [23–25].

Given the compatibility between CBT and new technologies, and the affinity for new technologies by youth, the integration of new technologies into CBT treatment of youth has been rapidly increasing [5, 7]. Applications have been developed for the treatment of a variety of disorders, including simple phobias, social anxiety disorder, generalized anxiety disorder, obsessive–compulsive disorder, encopresis, autism, eating disorders, depression, and substance abuse [26–39].

Mobile applications such as text messaging [i.e., short messaging services (SMS)] are particularly well suited for youth and can help clinicians implement CBT treatment more effectively through the use of homework reminders, real-time self-monitoring and between session communication and feedback [17]. Among mental health patients, text messaging is the most popular feature, and a higher percentage of mental health patients text compared to the general population [40, 41]. Self-monitoring in particular has been found to improve treatment outcomes, both by itself and when added to therapy [42, 43] and accounts for a significant portion of the variance in treatment outcomes [44]. Text messaging may help overcome non-compliance (a primary reason for lack of treatment efficacy) by enabling encouragement and support between sessions. Interacting with each adolescent on a daily basis to encourage compliance with homework assignments, evaluate progress, monitor side effects, etc., would be prohibitively expensive if clinicians were required to personally send and receive the messages themselves. Fortunately it is not necessary, given the demonstrated feasibility of automating those functions. There is a large body of literature on the efficacy of text messaging for improving heath behavior and treatment outcomes in other areas of health care (e.g., diabetes, asthma, hypertension, obesity), with positive outcomes in 93 % of the published studies [45]. Text messaging is also used in the treatment of psychiatric and substance use disorders in adults [46, 47]. Data on the use of SMS in the psychological treatment of youth and young adults are beginning to emerge [3, 48–52]. Teens have generally reacted favorably to use of SMS technology in treatment and prevention programs, with good compliance rates [22, 53].

In response to the National Institute of Mental Health’s call for research on the use of technology to facilitate the dissemination of evidence-based treatments [54], we developed a technology-enhanced intervention protocol to facilitate CBT treatment of adolescent depression. The program consists of three components, each using technology for a particular purpose: (1) online therapist training, (2) in-session use of tablets for teaching clients CBT concepts and skills, and (3) text messaging for between session homework reminders and self-monitoring. These three components help disseminate training to therapists, help therapists implement CBT with patients more effectively, and improve CBT treatment outcomes, respectively. The goal of this study was to evaluate the feasibility, user satisfaction, and effectiveness of this technology-enhanced approach for treating adolescent depression.

## Methods

### Clinicians

Eighteen licensed clinicians who work with depressed adolescents participated in the study. Clinicians were recruited through advertisements in professional journals and through direct mail (i.e., Psychology today listing of clinicians working with depressed adolescents). Clinicians came from 13 states and various disciplines, including social work (*n* = 7), clinical or counseling psychology (*n* = 9), educational psychology (*n* = 1) and behavioral mental health (*n* = 1). Fifteen had master’s degrees and three doctoral degrees. The mean age was 44.2 years (range 31–58 years, SD = 8.4), and 56 % (*n* = 10) were female. Thirteen were Caucasian, four African American, and one was multiracial. Fifteen (83 %) reported some prior exposure to CBT, primarily through group lectures (78 %). None were accredited or formally trained as CBT practitioners. Mean number of years working with adolescents was 12.2 (range 2–20 years, SD = 5.59).

### Patients

Sixty-five adolescents, aged 12–17 (mean age = 15.4, SD = 1.52) with a DSM-5 mood disorder [major depressive disorder (*n* = 31), persistent depressive disorder (*n* = 20), both major and persistent depressive disorders (*n* = 3), other specified depressive disorder (*n* = 6), unspecified depressive disorder (*n* = 5)] and a minimum score of 11 on the quick inventory of depressive symptomatology–adolescent-patient report (QIDS-A-Pat) (mean = 14.5, SD = 3.28, range 10–22) [55] were recruited. Subjects were excluded if they had bipolar disorder, severe conduct disorder, substance dependence, pervasive developmental disorders, thought disorder, severe suicidal/homicidal ideation or behavior requiring inpatient treatment. Diagnoses were determined via clinical interview using a DSM-5 symptom checklist. Non-English speakers and adolescents without daily access to a cell phone were also excluded. Patients represented diverse races and ethnicities, including Caucasian (*n* = 27), African American (*n* = 24), American Indian (*n* = 3), Asian (*n* = 1), Biracial (*n* = 5) and other (*n* = 5). Fifteen percent (*n* = 10) were Hispanic and two-thirds (*n* = 43) were female.

### Procedure

Clinicians were randomly assigned to have all their subjects receive either the technology-enhanced CBT intervention arm (CBT), or treatment as usual (TAU). Each clinician recruited four adolescents from their clinical practice who were initiating treatment for depression. Three clinicians dropped out of the study before completing enrollment and were replaced. Clinicians in the CBT arm completed a pre-test on CBT knowledge and then took the online tutorial on CBT treatment for adolescent depression. After completing the tutorial, clinicians took a post-test, then received an iPad containing a link to the online CBT interactive teaching materials and text-messaging system. A brief (1 h) orientation session was held with each clinician to review how to use the iPad for teaching CBT concepts to patients and for setting up text messages. Each patient was treated for 12 weeks, using the skills learned in the tutorial, and the in-session teaching tools. Individualized text messages were integrated into treatment. Clinicians in the TAU arm also recruited patients initiating treatment for depression from their clinical practice, and treated them for 12 weeks using usual care. After completing the study, clinicians in the TAU arm were offered access to the CBT training and intervention tools. Since both patients and therapists were considered research subjects, each signed informed consent statements approved by the Allendale Institutional Review Board. Patient flow and study completion rates by treatment arm are shown in Table 1.

**Table 1:** Patient recruitment and study completion by treatment arm

	Number enrolled	Number dropped, baseline to week 6	Week 6	Number dropped, week 6–week 12	Week 12
CBT	39	4	35	0	35
TAU	37	4	33	3	30
Total	76	8	68	3	65

### Description of the technology-enhanced CBT intervention

#### Online therapist training tutorial

The online training tutorial was developed as a way to address the critical shortage of clinicians trained in CBT, due in large part to a lack of training available [56, 57]. Putting the training online makes the training more accessible, cost-effective, and obviates the need for travel to one of the limited number of centers that offer CBT training. Trainees are not bound by time limitations, and can work at their own pace and schedule (a recent study found time and cost the strongest predictor of unwillingness to obtain training on empirically based treatments) [57]. The quality of the training is also enhanced using principles of instructional design to deliver multi-modal, interactive learning, both of which have been found to increase knowledge retention [58]. Standardizing the training helps insure the quality of the instruction, which is important as several studies have found that much of the CBT that is being delivered is not being administered properly [59, 60]. The tutorial was modeled after the cognitive behavior therapy manual used in the NIMH funded treatment of adolescents with depression study [61] and consisted of nine modules (overview, theoretical principals of CBT, explaining the nature of depression to Clients and the therapeutic relationship, explaining treatment rationale to clients, mood monitoring, goal setting, behavioral activation, problem solving, and cognitive restructuring). Trainees worked at their own pace, and could email us with any questions. The tutorial took about 5.5 h to complete (see http://telepsychology.net/OnlineAssessmentTools/Resources/Demo_Teen1/Story.html for examples of tutorial content). Animations, graphical illustrations, interactive exercises, and video illustrations of an expert clinician (Dr. Kennard) applying the techniques were used as teaching tools. Session agendas and a treatment protocol were provided to assist clinicians in treatment implementation.

#### Online interactive patient educational materials

The second component consisted of online instructional materials to help clinicians explain CBT concepts to patients. Patient understanding of treatment rationale and treatment concepts is a critical part of effective treatment, as the more sense a treatment makes to a client, the more likely they are to comply with it [62, 63]. In CBT, there is a collaborative relationship between the therapist and client, with the client seen as both capable of, and responsible for, change. To empower clients with the skills necessary for change, it is critical that both (the client and the depressed adolescents parents) have an basic understanding of the nature of depression, the CBT treatment rationale, and, CBT concepts and skills, such as mood monitoring, identifying and challenging automatic thoughts, and activity scheduling. Therapists typically teach this using a combination of verbal instruction and paper and pencil forms. We created a series of online, interactive education materials to (1) help novice CBT therapists structure sessions, (2) insure that the concepts are covered thoroughly and accurately, (3) engage and involve the youth and personalize the material, and (4) create personalized goals and homework assignments. For example, in teaching clients about automatic thoughts, the therapist first displays a hypothetical scenario on the tablet PC (in our case, an iPad), to teach the relationship between thoughts, emotions, and behaviors. The teen then generates two or three possible thoughts they might have in that situation and the different feelings associated with those thoughts. Once the concept is understood, i.e., that different thoughts lead to different feelings, the therapist goes through the process again using a situation from the client’s real life. Finally, homework is collaboratively set up, e.g., to monitor one’s mood and thinking at specific intervals during the day. In another example, the client may be learning problem-solving skills. In this case, the tablet plays a pre-recorded scenario of a typical teen problem, after which the client goes through the problem-solving process using the tablet. Finally, the process is repeated with a real-life problem the client has, followed by setting up problem-solving practice between sessions.

#### Interactive text messaging

The third part of the intervention consists of text messages the client receives between sessions to remind them of their homework goals, and to record results of homework practice (see http://www.telepsychology.net/CBTText_Default.aspx for an illustration). These are set up as the final step in the patient education process previously described. Typically, the client would receive two texts each day: a reminder text call in the morning and a text later in the day to record results. For example, if the goal was to increase pleasant activities, the morning text would say “remember to do at least one pleasant activity today’. Do you remember the activity you were going to do?” If they said no, they would receive a text back reminding them what the activity was. In the evening, they would receive a text asking them if they did the pleasant activity, get a reinforcing message if they did, and a text back asking them to describe what they did and how it affected their mood. If they did not do the activity, they would receive a text back saying “Making yourself do something when you do not feel like it is hard. Sometimes just doing something nice helps you feel better” followed by “Tell me what kept you from doing the activity today. We can talk about it next session.” A report of all texts sent and received is sent to the therapist for review with the client at their next session, as a way to process together how the homework went and to troubleshoot problems and reinforce learning. Timing and frequency of texts are determined collaboratively by the therapist and teen. For example, the teen may say that 9 pm is the best time to receive evening texts, as that is when he has some down time. Or a therapist may want to increase or decrease the frequency of mood monitoring, depending on the clinical status of the teen. Examples of texts are shown in Table 2.

**Table 2:** Examples of text messages and response options by therapeutic module

Module	Text message	Response options
Mood module: scheduled text	Please rate your mood right now, from 0 to 10. 0 would be a Very Bad Mood. 10 would be a Very Good Mood	0–10
Mood module: scheduled text	Please tell me why you gave that mood rating	Free text response
Goal setting: morning text	Hi. Remember your goal for the week is to {*insert sub*-*goal*}. Do you recall what step you were going to do today?	Yes/no
Goal setting: evening text 1	Hi. Did you {*insert first scheduled step*} today?	Yes/no
Goal setting: evening text 2	That’s great! Describe any problems you had, or any thing you’d like to discuss at our next session	Free text response
Goal setting: alternate evening text 2	Okay. Please describe any problems you had, or when you plan to take this next step	Free text response
Problem solving: morning text	Hi! This is a reminder to work on {*insert best solution*} this week. Do you recall the steps you were going to take?	Yes/no
Problem solving: evening text	“Were you able to do any steps today in your problem solving?”	Yes/no
Problem solving: evening text 2	Describe what steps you did and how successful you were	Free text response
Problem solving: alternate evening text 2	Ok. Describe any issues or problems you’d like to talk about at our next session	Free text response
Challenging unhelpful thoughts: morning text 1	Hi! Do you recall the process for challenging unhelpful thoughts?	Yes/no
Challenging unhelpful thoughts: morning text 2	“Great. I’ll check back with you tonight”	
Challenging unhelpful thoughts: alternative morning text 2	“Ok. Identify the event leading to unhelpful thought and the associated mood. Then think of alternative thoughts and how it will affect your mood”	
Challenging unhelpful thoughts: evening text 1	Do you want to thought challenge anything that happened today?	Yes/no
Challenging unhelpful thoughts: evening text 2	Ok, describe the event	Free text response
Challenging unhelpful thoughts : alternate evening text 2	“Ok, tell me how your day was today”	Free text response

### Outcome measures

#### Online tutorial

Effectiveness of the online tutorial in improving clinician’s knowledge of CBT concepts was evaluated using a 49-item multiple choice pre- and post-test covering the tutorial content. The test had good internal consistency reliability (coefficient alpha = 0.821). Technical feasibility of the tutorial was evaluated with the system usability scale (SUS) [64, 65]. The SUS is a reliable, well-validated 10-item scale designed to evaluate the usability and user satisfaction with web-based applications and other technologies. The SUS has good internal consistency reliability (coefficient alpha: *r* = 0.86 in our sample) in assessing usability across diverse types of user interfaces (e.g., web, interactive voice response, cell phone, etc.) It provides quantitative feedback on a 0–100 scale. In a cross-validation study of the SUS using an anchored adjective scale, systems with “Good” usability had mean score of 71.4. [66] This criterion was used for successful system design in the current study. In addition to the SUS, ratings were also obtained on whether the stated learning objectives of the tutorial were met, and a set of questions evaluating satisfaction with the clinical content of the tutorial.

#### Online teaching materials and text messaging

Technical feasibility with the online teaching materials and text-messaging system was evaluated with the SUS. Open-ended feedback was also solicited on user satisfaction with the system from both clinicians and patients.

#### Clinical outcomes

Clinical outcome measures were obtained at the end of 6 and 12 weeks of treatment. The primary clinical outcome measure was pre-to-post treatment changes in patient ratings of depression on the quick inventory of depressive symptomatology–adolescent version (QIDS-A-Pat) [55]. Secondary outcomes included clinician global ratings of improvement (CGI-I) and severity (CGI-S) [67], and clinician and patient ratings on the therapeutic alliance scale for adolescents (TASA) [68].

#### Statistical analyses

Categorical and ordinal variables, such as gender and percent responders were tested by Chi-square tests of distributional independence. Interval and ratio level measurements, such as age, and depression severity scores were compared with two-tailed, between group *t* tests for equivalence of means. When the sample size in each of two groups is 32, a 0.05 level Chi-square test will have power of 0.7–0.97 to distinguish between the groups when the proportions in the two categories are characterized by effect sizes of 0.1 to 0.25. Samples of 32 per group have statistical power of 0.50–0.88 to detect moderate to large mean differences (effect sizes of 0.5–0.8) in two group *t* tests using two-sided alphas of 0.05. The sample size estimate was based on the QIDS-A-Pat.

## Results

### Clinicians and patients

There were no significant differences between clinicians randomized to CBT and TAU in terms of age [t(16) = 0.42, *p* = 0.678), gender (X^2^(1) = 1.90, *p* = 0.168], or years’ experience [*t*(16) = 0.10, *p* = 916]. There were also no significant differences between patients in the CBT and TAU arms on age [*t*(63) = 0.076, *p* = 0.940], gender (X^2^(1) = 0.94, *p* = 0.432), or baseline depression severity (QIDS-A-Pat) [*t*(63) = 0.27, *p* = 0.787).

### Online tutorial

#### Increase in didactic knowledge

We examined changes in scores on the 49-item pre-and post-tests of knowledge of CBT concepts covered in the tutorial. A significant increase was found in the number of correct items from the pre-test (24.4, SD = 4.42) to the post-test (33.9, SD = 5.11), t(8) = 7.02, *p* < 0.001.

#### Learning objectives

Twenty-three learning objectives were identified a priori as learning goals for the online tutorial (Table 3). After completing the tutorial, 97 % of the learning objectives were rated as met. The mean rating of how much they learned as a result of taking the tutorial was 4.4 (rated on a 1–5 scale (1 = very little and 5 = a great deal).

**Table 3:** Learning objectives: CBT tutorial

After completing the tutorial do you feel able to:
Module 1. Theoretical principles of CBT
Describe the core concepts behind Becks Cognitive Theory of Depression, Learned Helplessness, & Social learning
Describe the main idea behind Social Learning Theory
Describe the nature of therapeutic relationship in CBT
Module 2. Explaining the nature of depression to clients
Provide key information to clients on the nature of depression
Module 3. Explaining treatment rationale to clients
Explain the rationale underlying CBT treatment to clients
Help clients identify initial treatment goals
Explain CBT session structure and format to clients
Module 4. Mood monitoring
Explain the rationale for mood monitoring to clients
Teach clients how to monitor their mood
Develop a plan for mood monitoring for the teen to use before the next session
Module 5. Goal setting
Teach clients the basic principles of goal setting
Explain the rationale for breaking down goals into sub-goals, making them specific, and attainable
Help clients set long- and short-term treatment goals
Module 6. Behavioral activation
Explain the rationale for behavioral activation
Teach clients the skill of activity scheduling
Teach clients the skill of increasing pleasant activities
Module 7. Problem solving
Explain the rationale for problem solving to clients
Describe the steps in problem solving
Describe emotional barriers to problem solving and how to deal with them
Module 8. Cognitive restructuring
Explain What Automatic Thoughts are to clients (called “unhelpful thoughts”)
Teach clients to identify their unhelpful thoughts
Teach clients how to replace automatic thoughts with more helpful and realistic thoughts through
Teach clients how to use the three and 5 column mood monitoring forms for identifying and challenging unhelpful thoughts

#### User satisfaction: technical aspects

The means score on the SUS for the online tutorial was 78.4 (SD = 20.44) (Table 4). This corresponds to a score of good user satisfaction on the SUS. Mean global rating of user-friendliness (rated scale range from 1 (worst imaginable) to 7 (best imaginable)) was 5.6, which is halfway between “good” and “excellent”.

**Table 4:** System usability scale scores for the online tutorial and teaching/text system

Adjective	SUS cutoff score	Mean (SD) in the current study
Worst imaginable	12.5	
Awful	20.3	
Poor	35.7	
Ok	50.9	
Good	71.4	78.4 (20.4)-Tutorial
		84.4 (13.8)-Teaching/text
Excellent	85.5	
Best imaginable	90.0	

From Bangor et al. [66]

#### User satisfaction: clinical content

Descriptive statistics were obtained on user satisfaction with the online tutorial (Table 5). All that subjects agreed or strongly agreed that the material was presented in an interesting manner, was clearly presented and easy to understand, and was useful and relevant to treating adolescent depression. All would recommend the online tutorial to others.

**Table 5:** Mean satisfaction ratings on tutorial scale clinical content

Item	Mean (SD)
1. The material was presented in an interesting manner	3.6 (0.55)
2. The concepts were clearly presented and easy to understand	3.7 (0.49)
3. The content was useful and relevant to treating adolescent depression	3.7 (0.48)
4. I would recommend this course to others	3.7 (0.48)
5. Overall, how satisfied were you with this module?	3.7 (0.48)

Items 1–4, scale = 1 = strongly disagree, 2 = disagree, 3 = agree, 4 = strongly agree

Item 5:1 = very dissatisfied, 2 = dissatisfied, 3 = satisfied, 4 = very satisfied

### Online teaching materials and text messaging

#### User satisfaction: clinicians

The means score on the SUS for the online CBT teaching materials and text-messaging system was 84.4 (SD = 13.80). This corresponds to a score between good and excellent. Ratings on individual SUS items are presented in Table 6. The mean rating for all items was between “agree” and “strongly agree”. Clinicians found the system ‘user friendly’ in terms of understanding how to utilize the system for teaching CBT skills, setting up text messages, and receiving text reports.

**Table 6:** Mean ratings on system usability scale items: online teaching materials and text messaging

Item	Mean (SD)
1. I would like to use this system frequently	3.4 (0.90)
2. I found the system unnecessarily complex	3.3 (0.85)
3. I thought the system was easy to use	3.3 (0.85)
4. I think I would need the support of a technical person to be able to use this system	3.2 (0.87)
5. I found the various functions in this system were well integrated	3.6 (0.66)
6. I thought there was too much inconsistency in this system	3.5 (0.58)
7. I would imagine that most people would learn to use this system very quickly	3.2 (0.75)
8. I found the system very cumbersome to use	3.5 (0.65)
9. I felt confident using the system	3.5 (0.53)
10. I needed to learn a lot of things before I could get going with this system	3.3 (0.69)

Scale range 1 = strongly disagree, 2 = disagree, 3 = neutral, 4 = agree, 5 = strongly agree

Items 2, 4, 6, 8 and 10 are reverse scored

#### User satisfaction: patients

Feedback was also solicited from adolescents on how helpful the teaching and text message system was. Eighty-five percent of patients felt the teaching materials presented on the iPad during sessions were helpful in learning new skills, 90 % felt the text messages between sessions were helpful, and 95 % said reviewing their text message responses on their homework and mood at the next session with their therapist were helpful. All patients said they would be willing to use text messaging again to communicate their feelings to their clinician between sessions.

### Clinical outcomes

Both treatment groups significantly improved with treatment, with mean improvements on the QIDS-A of 6.09 (SD = 4.26) and 5.73 (SD = 4.71) for the CBT and TAU groups [*t*(34) = 8.453, *p* < 0.001 and *t*(29) = 6.67, *p* < 0.001 respectively]. Clinical outcome measures comparing the CBT and TAU groups are presented in Table 7. Therapist ratings of the therapeutic alliance (TASA) were significantly higher in the CBT intervention arm than in the TAU arm, *t*(131) = 4.03, *p* = 0.001. Measures of symptomatic improvement were greater on all other outcome measures for the CBT arm; however, none reached statistical significance. Effect sizes (Cohen’s d) [69] ranged from small (QIDS-A) to large (TASA).

**Table 7:** Clinical outcome measures: CBT vs. TAU

	QIDS-A: mean change	CGI-S: percent normal or borderline depressed at week 8	CGI: percent rated much or very much improved at week 8	Therapeutic alliance rating: patients	Therapeutic alliance rating: clinicians
CBT	6.09	51.4 %	71.4 %	62.8	65.3
TAU	5.73	46.7 %	60 %	60.02	58.4
Diff	0.35			2.8	6.9
P	0.753	0.825	0.332	0.03	0.001
Effect size	0.08	0.11	0.28	0.31	0.70
95 % Confidence interval, effect size		−0.433, 0.6436	−0.289, 0.852	−0.0273, 0.0306	0.7002, 0.3496

### Text messaging

A total of 9,613 text messages requiring a response were sent. Of these, 3658 (38.1 %) were responded to. The correlation between improvement on the QIDS-A and percent of texts responded to was not significant (*r* = 0.165, *p* = 0.343).

### Dropout rate

Seven subjects dropped out prior to week 12 in the TAU arm, compared to 4 subjects in the CBT arm.

## Discussion

Results of this study provide support for the feasibility of this technology-enhanced CBT Intervention as a means of improving CBT treatment of adolescent depression. User satisfaction, a critical component of feasibility, was high for both adolescents and therapists on all components. The program was successful in increasing therapists’ knowledge of CBT concepts and principles. Teens found the online teaching tools useful for learning CBT concepts and skills. They also found the text messaging between sessions helpful, particularly for reviewing work done between sessions with their therapist. All teens indicated they would be willing to use the system again. Rather than put a barrier between the teen and the therapist, the technology improved the therapeutic bond, a critical factor in treatment outcomes. Improving the therapeutic relationship may help to keep teens in treatment, a critical factor for successful outcomes.

From a system delivery perspective, the use of this technology-enhanced intervention is designed to augment rather than replace existing one to one clinician care. As such it is not a low intensity intervention (i.e., an intervention designed to limit therapist time) [70]—and keeps the same number and lengths of session as usual. This approach contrasts recent “stepped-care” models of treatment, which start with the least restrictive treatment with minimal therapist support. Future research can examine the use of this (and similar) technologies within a stepped-care model. This could include examining factors such as length of treatment, use of online self-help combined with therapist and non-therapist support, both with and without text-messaging augmentation.

Effect sizes on the clinical outcomes in the current study were small to medium. According to Cohen, a small effect size is one in which there is a real effect, but can only be seen through careful study. The current study used community clinicians (vs. academic research centers) to see how well the intervention works in a sample of community therapists that not had formal training in CBT. Taken in this light, small effects are encouraging. As the training continues to be evaluated and refined, the impact of additional follow-up training, or live applied training may further improve results. Prior studies with remote CBT training found the addition of live remote observation through a videoconference of trainees conducting CBT, with immediate feedback in real time significantly improved clinical skills [16]. The addition of this applied training component may have improved clinical outcomes. A follow-up study is underway to examine the impact of the addition of live training on post-training treatment outcomes with community patients.

The current program utilized technology to integrate three components as part of a single intervention: therapist training, client education, and treatment implementation and outcomes. As the use of technology continues to be adopted and integrated into clinical treatment, more empirical evidence will help shed light on which components are useful and under what circumstances. At a minimum, the current intervention helps address the critical shortage of training on empirically based treatments. The potential ways in which technology such as text messaging and use of interactive educational tools can enhance treatment are at the start of a new era of clinical research. New possibilities are rapidly emerging, and to some extent, are outpacing our ability to empirically evaluate these new innovations [71]. Some recent data suggest that the explosion of mental health apps has resulted apps of poor quality, or apps that do not reflect clinical practice guidelines or evidence-based practices [72, 73]. However, while presenting many challenges, they also present exciting opportunities. Continued research should continue to generate empirical data to help guide both clinical practice as well as future research in this area.

## Conclusion

In the current study, a technology-enhanced CBT Intervention was effective in improving symptoms of depression in adolescents. User satisfaction with the technology was high for both therapists and patients. The therapeutic alliance was stronger in the cohort receiving the technology-enhanced intervention. Effect sizes comparing clinical outcomes between CBT and TAU were small.
