# Erratum to: Integrating technology into cognitive behavior therapy for adolescent depression: a pilot study

**DOI:** 10.1186/s12991-015-0089-4

**Published:** 2016-01-15

**Authors:** Kenneth A. Kobak, James C. Mundt, Betsy Kennard

**Affiliations:** Center for Telepsychology, 22 North Harwood, Madison, WI 53717 USA; UT Southwestern Medical Center, 5323 Harry Hines Boulevard, Dallas, TX 75390 USA

## Erratum to: Ann Gen Psychiatry (2015) 14:37 DOI 10.1186/s12991-015-0077-8

Following publication of the original version [1] of the article in the *Annals of General Psychiatry*, it was brought to our attention that there was an error in Table 7. It should say ‘week 12’ above columns 2 and 3, instead of ‘week 8’.

**Table 7:** Clinical outcome measures: CBT vs. TAU

	QIDS-A: mean change	CGI-S: percent normal or borderline depressed at week 12	CGI: percent rated much or very much improved at week 12	Therapeutic alliance rating: patients	Therapeutic alliance rating: clinicians
CBT	6.09	51.4 %	71.4 %	62.8	65.3
TAU	5.73	46.7 %	60 %	60.02	58.4
Diff	0.35			2.8	6.9
P	0.753	0.825	0.332	0.03	0.001
Effect size	0.08	0.11	0.28	0.31	0.70
95 % Confidence interval, effect size		−0.433, 0.6436	−0.289, 0.852	−0.0273, 0.0306	0.7002, 0.3496
